# Exploring the unique function of imprinting control centers in the PWS/AS-responsible region: finding from array-based methylation analysis in cases with variously sized microdeletions

**DOI:** 10.1186/s13148-019-0633-1

**Published:** 2019-02-28

**Authors:** Keiko Matsubara, Masatsune Itoh, Kenji Shimizu, Shinji Saito, Keisuke Enomoto, Kazuhiko Nakabayashi, Kenichiro Hata, Kenji Kurosawa, Tsutomu Ogata, Maki Fukami, Masayo Kagami

**Affiliations:** 1 Department of Molecular Endocrinology, National Center for Child Health and Development, 2-10-1 Ohkura, Setagaya-ku, Tokyo, 157-8535 Japan; 2 Department of Pediatrics, Kanazawa Medical University, Kanazawa, 920-1192 Japan; 3 Division of Medical Genetics, Saitama Children’s Medical Center, Saitama, 330-8777 Japan; 4 Department of Pediatrics and Neonatology, Nagoya City University Graduate School of Medical Sciences, Nagoya, 467-8601 Japan; 5 Enomoto Children’s Clinic, Moriya, 302-0127 Japan; 6 Department of Pediatrics and Developmental Biology, Tokyo Medical and Dental University Graduate School, Tokyo, 113-8510 Japan; 7 Department of Maternal-Fetal Biology, National Center for Child Health and Development, Tokyo, 157-8535 Japan; 8 Division of Medical Genetics, Kanagawa Children’s Medical Center, Yokohama, 232-8555 Japan; 9 Department of Pediatrics, Hamamatsu University School of Medicine, Hamamatsu, 431-3192 Japan

**Keywords:** 15q11–13, Prader-Willi syndrome, Angelman syndrome, Genome-wide methylation study, Deletion

## Abstract

**Background:**

Human 15q11–13 is responsible for Prader-Willi syndrome (PWS) and Angelman syndrome (AS) and includes several imprinted genes together with bipartite elements named AS-IC (imprinting center) and PWS-IC. These concertedly confer allele specificity on 15q11–13. Here, we report DNA methylation status of 15q11–13 and other autosomal imprinted differentially methylated regions (iDMRs) in cases with various deletions within the PWS/AS-responsible region.

**Methods:**

We performed array-based methylation analysis and examined the methylation status of CpG sites in 15q11–13 and in 71 iDMRs in six cases with various microdeletions, eight cases with conventional deletions within 15q11–13, and healthy controls.

**Results:**

We detected 89 CpGs in 15q11–13 showing significant methylation changes in our cases. Of them, 14 CpGs in the *SNORD116*s cluster presented slight hypomethylation in the PWS cases and hypermethylation in the AS cases. No iDMRs at regions other than 15q11–13 showed abnormal methylation.

**Conclusions:**

We identified CpG sites and regions in which methylation status is regulated by AS-IC and PWS-IC. This result indicated that each IC had unique functions and coordinately regulated the DNA methylation of respective alleles. In addition, only aberrant methylation at iDMRs in 15q11–13 leads to the development of the phenotypes in our cases.

**Electronic supplementary material:**

The online version of this article (10.1186/s13148-019-0633-1) contains supplementary material, which is available to authorized users.

## Introduction

**Fig. 1:**
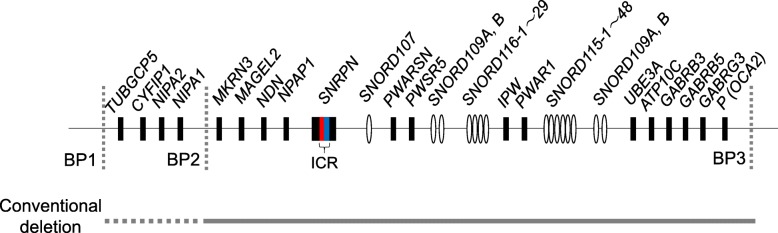
Schematic diagram of the PWS/AS region. Horizontal gray line and dotted line indicate the range of conventional large deletion bounded by breakpoint (BP) 1 or 2 and BP 3. snoRNA genes are shown in ovals. Not all genes of the locus are shown, and the map is not to scale

The chromosomal region 15q11–13 includes several genes showing monoallelic expression in a parent-of-origin-specific manner [1]. This region is responsible for Prader-Willi syndrome (PWS, OMIM #176270) and Angelman syndrome (AS, OMIM #105830). PWS is characterized by hypotonia during infancy, developmental delay, hyperphagia followed by morbid obesity, and cognitive impairment [2], and AS is associated with severe developmental delay, ataxia, and recurrent seizures [3]. Approximately 70% of PWS and AS patients have 5–7 Mb deletions affecting the 15q11–13 imprinted region (Fig. 1). Recently, it has been reported that deletion involving the *SNORD116* gene, which is one of the C/D box small nucleolar RNAs (snoRNAs), causes the phenotypes of PWS [4–8]. AS phenotypes arise from the loss of expression or function of the *UBE3A* gene, which is expressed on the maternally derived allele in mature neurons [3].

The imprinting control region (ICR) conferring parent-of-origin identity of the genes on 15q11–13 was defined according to the smallest region of overlap (SRO) found in PWS or AS individuals with rare atypical microdeletions [9]. The ICR on 15q11–13 consists of bipartite DNA elements named AS-IC (imprinting center) and PWS-IC [10]. The PWS-IC is a 4.1-kb region, which spans the *SNURF/SNRPN* promoter and exon 1, and includes an imprinted differentially methylated region (iDMR), which is maternally methylated and paternally unmethylated. The AS-IC is an 880-bp sequence located ~ 35 kb centromeric of the PWS-IC and does not include iDMR. It is known that PWS-IC and AS-IC cooperate in regulating epigenetic status and allele-specific gene expression at this locus [1].

Recent advancement in methylation analysis allows genome-wide methylation studies, and it detected novel iDMRs located in regions near the *SNURF/SNRPN* gene and several CpG sites in the *SNORD116s* cluster showing a slight tendency for preferential paternal methylation [11]. The regulatory mechanism in DNA methylation of those imprinted regions by ICR has not yet been elucidated precisely.

In this study, we performed an array-based DNA methylation analysis in the cases with various deletions involving the PWS/AS region and examined the methylation status of CpG sites in 15q11–13 and in 71 iDMRs at regions other than the PWS/AS region to clarify the regulatory mechanism for DNA methylation in the 15q11–13 imprinted region.

## Results and discussion

Six cases with atypical microdeletions in the 15q11–13 region (cases 1–6) and four with PWS and four with AS due to conventional large deletions (LD) in 15q11–13 (PWS_LD_ and AS_LD_, respectively) were enrolled in this study (Table 1 and Additional file 1: Supplementary document).

**Table 1:** Clinical information, deletion range, and methylation status at *SNRPN*-DMR in cases enrolled in this study

Cases		Phenotype	Sex	Age^a^	Breakpoint (approximate size)	Methylation status at *SNRPN-*DMR^d^
Case 1		PWS	Male	2 mo	Chr15:25,150,978-25,225,535 (75 kb)^b^	Hypermethylated
Case 2		Healthy carrier (father of case 1)	Male	36 yr		Hypomethylated
Case 3		PWS	Female	3 yr	Chr15:25,216,569-25,415,670 (200 kb)^b^	Normal
Case 4		AS	Female	3 yr	Chr15:25,126,774-25,168,037 (41 kb)^b^	Hypomethylated
Case 5		AS	Male	4 yr	Chr15:25,164,853-25,168,575 (3.7 kb)^b^	Hypomethylated
Case 6		Healthy carrier (mother of case 5)	Female	36 yr		Normal
PWS_LD_	PWS_LD_-1	PWS	Male	0 mo	BP2–3 (5.5 Mb)^c^	Hypermethylated
	PWS_LD_-2	PWS	Male	2 mo	BP1–3 (6 Mb)^c^	Hypermethylated
	PWS_LD_-3	PWS	Male	3 yr	BP1–3 (6 Mb)^c^	Hypermethylated
	PWS_LD_-4	PWS	Male	8 yr	BP1–3 (6 Mb)^c^	Hypermethylated
AS_LD_	AS_LD_-1	AS	Female	9 yr	BP2–3 (5.5 Mb)^c^	Hypomethylated
	AS_LD_-2	AS	Female	9 yr	BP2–3 (5.5 Mb)^c^	Hypomethylated
	AS_LD_-3	AS	Male	2 yr	BP1–3 (6 Mb)^c^	Hypomethylated
	AS_LD_-4	AS	Female	1 yr	BP1–3 (6 Mb)^c^	Hypomethylated

*PWS* Prader-Willi syndrome, *AS* Angelman syndrome, *DMR* differentially methylated region, *LD* large deletion, *BP* breakpoint

^a^Age at sample collection (*mo* months, *yr* years)

^b^The breakpoints were estimated according to the results of aCGH

^c^The breakpoints were estimated according to the results of methylation-specific multiplex ligation-dependent probe amplification (MS-MLPA). The locations of BPs are shown in Additional file 2: Figure S1

^d^Methylation status were examined by MS-MLPA

### Deletion size and breakpoint

**Fig. 2:**
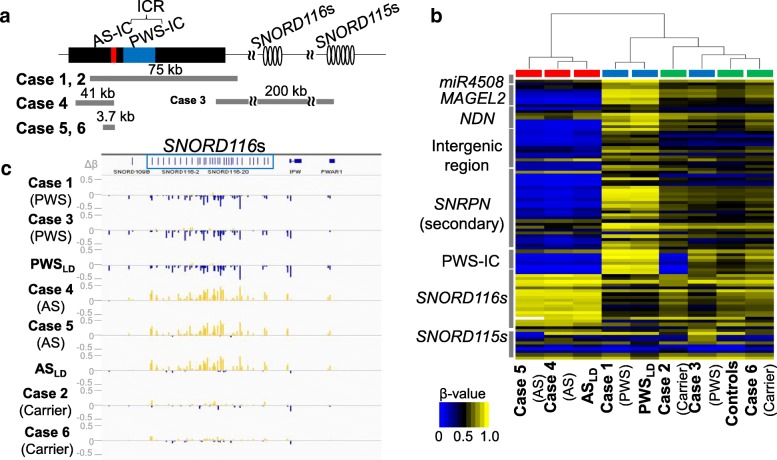
Genomic organization of the PWS/AS locus and alteration in DNA methylation of CpG sites in 15q11–13. **a** Summary of the loci and the approximate sizes of the deleted regions in cases 1–6. **b** Unsupervised hierarchical clustering and heat map of the 450k methylation data of 89 probes with |Δβ| > 0.2 and FDR *p* values < 0.01 between at least 1 case and controls are shown. The cases with AS phenotype are shown in red boxes, those with PWS phenotype in blue boxes, and the carriers and normal controls in green boxes. Blue and yellow colors indicate 0 and 1 methylation, respectively. The *y*-axis represents the names of iDMRs which had been known to be differentially methylated previously. Not all names of the iDMRs are shown. **c** The differences in *β* values (Δ*β*) of probes located in the *SNORD116s* and *115s* clusters between each case and controls are shown using IGV (Integrated Genome Viewer, http://software.broadinstitute.org/software/igv/). The probes showing “hypermethylated” in cases are represented by yellow vertical bars, and those showing “hypomethylated” by blue ones. ICR, imprinting control region; IC, imprinting center; kb, kilobase

Custom-built array CGH revealed a copy number loss in each case (Fig. 2a and Additional file 2: Figure S1). This array contained approximately 38,000 probes for 15q11–q13 encompassing the imprinted region and ~ 10,000 reference probes for other chromosomal regions (4×180K format, Design ID 032112) (Agilent Technologies, Palo Alto, CA, USA). The detailed information of probes contained in this custom-built array is shown in Additional file 3: Table S1. Cases 1 and 2 had a deletion involving both AS-IC and PWS-IC, case 3 had a deletion involving only the *SNORD116s* cluster, and cases 4–6 had a deletion involving only AS-IC.

### Difference in the DNA methylation status between each case with various microdeletions and controls

We focused on the DNA methylation status at CpG sites on autosomal chromosomes in two groups (groups 1 and 2) and extracted methylation data at 1335 probes located in CpG sites at the 15q11–13 PWS/AS region (group 1, Additional file 4: Table S2) and at 863 probes in the iDMRs at regions other than the PWS/AS region (group 2, Additional file 5: Table S3)**.**

### CpG sites in 15q11–13 (probes in group 1)

We identified 89 probes in group 1 showing significant differences in DNA methylation in at least 1 case against normal controls (Fig. 2b and Additional file 6: Table S4). Most probes located in the *miR4508*-PWS-IC region showed hypermethylation in case 1 and PWS_LD_ and hypomethylation in cases 4 and 5 and AS_LD_, respectively. Case 3 showed normal methylation status in these sites. Hierarchical clustering of the methylation status of these 89 CpGs showed that all cases other than case 3 with the *SNORD116*s deletion were classified into subgroups according to their phenotypes (Fig. 2b). Case 3 was clustered closer to the normal controls.

In case 2, who was the father of case 1, probes in his deleted region in PWS-IC showed hypomethylation; however, the remaining probes showed normal methylation status. In case 6, who was the mother of case 5, no CpG with aberrant methylation was found.

In addition, 14 probes in the *SNORD116s* cluster presented slight hypomethylation in the cases with PWS phenotype (cases 1and 3 and PWS_LD_) and hypermethylation in the cases with AS phenotype (cases 4 and 5 and AS_LD_) (Fig. 2c).

### iDMRs at regions other than the PWS/AS region (probes in group 2)

Additional file 7: Table S5 shows the results of the methylation analyses for 71 iDMRs including 828 probes in group 2 between each of cases 1–6 and normal controls. We detected no iDMR with an abnormal methylation pattern.

**Fig. 3:**
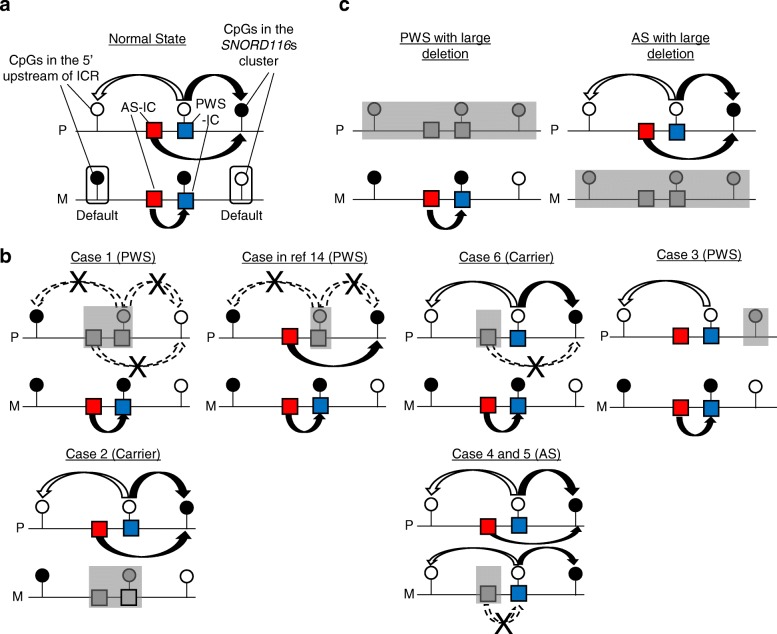
Hypothetical model for the regulation of the DNA methylation at the PWS/AS region by AS-IC and PWS-IC. Circles denote CpGs showing differential methylation patterns in a parent-of-origin-specific manner. Solid circles represent methylated CpGs, and the open ones unmethylated CpGs. P denotes paternally inherited allele, and M maternally derived allele. Deleted regions are shown by gray boxes in each case. **a** Mechanism regulating the DNA methylation at the PWS/AS region in normal individuals. On the P allele, CpGs in PWS-IC and the promoter of paternally expressed genes in the 5′ upstream region of ICR are unmethylated, and CpGs located sparsely in the *SNORD116*s cluster are methylated. The unmethylated PWS-IC on the P allele is thought to lead to CpGs with a paternal methylation pattern at CpGs in both the upstream region (unmethylated, shown by white arrows) and the *SNORD116*s cluster (methylated, shown by black arrows). AS-IC on the P allele methylated CpGs in the *SNORD116*s cluster. On the M allele, PWS-IC and CpGs in iDMRs of upstream imprinted genes are methylated, and CpGs in the *SNORD116*s cluster are unmethylated. AS-IC on the M allele methylated PWS-IC. Maternal methylation pattern (methylated in the upstream region and unmethylated in the *SNORD116*s cluster) is the default state. **b** Regulation of the DNA methylation in cases with various microdeletions involving AS-IC, PWS-IC, or *SNORD116*s cluster. The cases with deletions in the paternally inherited allele are shown in the upper part and those in the maternally derived allele in the lower part. In the second left case with a deletion involving only PWS-IC in the paternally inherited allele, the methylation pattern in the *SNORD116*s cluster is depicted according to the results of a previous report [14]. **c** DNA methylation pattern in cases with large deletions involving the entire PWS/AS region. AS-IC and PWS-IC seemed to control DNA methylation status cooperatively or separately at the PWS/AS region

In this study, we examined the methylation status of CpG sites in 15q11–13 and in 71 iDMRs at regions other than the PWS/AS region using methylation array for the cases with various deletions involving the PWS/AS region. This study provides several notable findings. First, we clarified CpG sites and regions in which the methylation status was regulated by AS-IC and PWS-IC in 15q11–13. Most abnormally methylated CpG sites in cases with deletions were located within or very close to the regions reported in previous studies [11–13]. Based on the results from our patients and a previously reported PWS patient with a microdeletion involving only PWS-IC, we present a hypothetical model for the regulation of DNA methylation at the 15q11–13 imprinted region in Fig. 3. We suggest regulatory mechanisms of this region as follows: (1) maternal pattern (methylated) at CpGs in the upstream region of the ICR was the default state (see normal state and case 2), (2) AS-IC was required for the methylation of PWS-IC on the maternally derived allele (compare case 6 with cases 4 and 5), (3) the unmethylated PWS-IC led to the unmethylated status of CpGs in the upstream of the ICR even on the maternally derived allele (see cases 4 and 5), and (4) the methylation patterns of several CpGs in the *SNORD116*s clusters were also regulated by both AS-IC and PWS-IC. Several CpGs in the *SNORD116*s cluster were preferentially methylated on the paternally inherited allele (Fig. 2c) [11]. The methylation pattern in the *SNORD116*s cluster was different between case 1 and the PWS case lacking only PWS-IC [14]. These results indicate that both AS-IC and unmethylated PWS-IC on the paternally derived allele function independently as regulators of the methylation status of CpGs in the *SNORD116s* cluster. Furthermore, several CpGs in the *SNORD116*s cluster were methylated in cases 4 and 5 on the maternally derived allele. This result indicates that unmethylated PWS-IC was needed for the methylation of the CpGs in the *SNORD116*s cluster independently on the parental origin. In the cases with large deletions involving the entire region of 15q11–13 (Fig. 3c), abnormal methylation patterns were simply due to the loss of the paternally or maternally derived allele in PWS or AS patients, respectively.

Second, this study demonstrated that PWS/AS case with various deletions had normal methylation status in known iDMRs at regions other than 15q11–13. This result indicates that the development of phenotypes in our cases was not caused by aberrant methylation changes at iDMRs at regions other than 15q11–13. In addition, our study and previous studies using other methylation analyses showed a normal methylation pattern at PWS-IC in patients with deletions only including the *SNORD116*s cluster [4–7]. It remains to be clarified how the deletion of *SNORD116*s contributes to the development of PWS phenotypes. Previous studies reported several findings regarding long non-coding RNAs (sno-lncRNAs) including *SNORD116*s: (1) some functional units, 116 host genes (116HG) and snoRNA-related sno-lncRNAs, were processed from the *SNORD116*s cluster in human tissues or cells [15, 16], (2) Fox-family splicing regulators are bound to sno-lncRNAs and altered splicing patterns of genes are related to neuronal development in human ES cells [17], and (3) there was a *Snord116*-dependent diurnal rhythmic DNA methylation in the mouse cortex [18]. Thus, the loss of the expression of *SNORD116*s may lead to PWS-relevant phenotypes, such as abnormalities of energy metabolism and diurnal rhythm [17, 18].

It remains to be elucidated how AS-IC and PWS-IC establish the allele-specific methylation patterns in neighboring CpGs. Recently, it was reported that transcripts from an oocyte-specific promoter in AS-IC are needed for the acquisition of maternal DNA methylation patterns in PWS-IC in human oocytes [19]. However, there has apparently been no study examining the direct association between AS-IC and the *SNORD116*s cluster, although unmethylated PWS-IC on the paternal allele physically interacts with paternally expressed genes in the upstream region of ICR [14]. In addition, how unmethylated PWS-IC establishes paternal methylation patterns at CpGs in the PWS/AS region in *cis* and how PWS-IC interacts with CpG sites at the *SNORD116*s cluster on paternally derived alleles remain unknown.

In summary, we performed an array-based methylation study in cases with various-sized microdeletions in the PWS/AS region on maternally or paternally derived chromosome 15. We identified CpG sites and regions in which the DNA methylation status is regulated by ICR. Our results enabled us to speculate on the regulatory mechanism for the establishment of the methylation status of local CpG sites or regions in 15q11–13. Moreover, we demonstrated that the development of phenotypes in the PWS/AS patients with deletions encompassing ICR and/or the *SNORD116s* cluster was not mediated by aberrant methylation changes at iDMRs at regions other than the PWS/AS regions. Further investigation will be necessary to fully elucidate how paternal deletion of *SNORD116*s causes PWS phenotypes.

## Materials and methods

### Subjects

Six cases with atypical microdeletions in the 15q11–13 region (cases 1–6) and four with PWS_LD_ and four with AS_LD_ due to conventional large deletions were enrolled in this study (Figs. 1 and 2a, Table 1, Additional file 1: Supplementary document, and Additional file 8: Figure S2). Genomic DNAs from leukocytes in these cases and healthy controls were utilized for this study. Methylation-specific multiplex ligation-dependent probe amplification (MS-MLPA; ME030, MRC-Holland, Amsterdam, Netherlands) was performed in all cases to examine the deleted region and DNA methylation status in 15q11–13 (data not shown). All cases showed normal karyotype. Clinical manifestations of cases are shown in the supplementary document. Healthy children (*n* = 11) and adults (*n* = 24) were involved as normal controls.

### Copy number analysis

We designed a custom-built array-based comparative genomic hybridization (aCGH) and utilized this aCGH to narrow down the deleted regions in cases 1–6 (4×180K format, Design ID 032112, Agilent Technologies, Palo Alto, CA, USA). The detailed information of probes contained in this array is shown in Additional file 3: Table S1. The procedure was as described in the manufacturer’s instructions.

### Comprehensive methylation analysis using the HumanMethylation450 BeadChip

We performed methylation assays on cases with various deletions together with healthy controls using the Infinium HumanMethylation450 BeadChip (HM450k, Illumina, Inc., San Diego, CA, USA). Detailed procedures are shown in Additional file 1: Supplementary document.

### HM450k data processing

The HM450k data were processed using the “ChAMP” R package version 1.10.0 [20]. Detailed algorithms of data pre-processing are available in the supplementary document and Additional file 9: Figure S3. We extracted the methylation data at 1335 probes located in the CpG sites at the 15q11–13 PWS/AS region (group 1, Additional file 4: Table S2) and at 863 probes in the iDMRs at regions other than the PWS/AS region (group 2, Additional file 5: Table S3) [11, 12]. Of note, 161 probes in group 1 presented with allele-specific methylation status according to the parental origin [11–13].

Before making comparisons of the methylation status between each single case with microdeletion (cases 1–6), patients with PWS_LD_ or with AS_LD_, and controls, we excluded five probes in group 1 (cg26889953, cg1789680, cg09873524, cg26955196, and cg11826104) showing differential methylation status between child and adult controls from the group 1 list for further analysis (Additional file 1: Supplementary document and Additional file 9: Figure S3). There was no probe showing a different methylation status between child and adult controls in group 2.

Subsequently, we compared the methylation status between each single case (cases 1–6), patients with PWS or with AS due to large deletions, and normal controls. The methylation level at each probe was represented by *β* values ranging from 0 (completely unmethylated) to 1 (completely methylated). The differences in DNA methylation (Δ*β*) were calculated by the subtraction of the *β* value in each case from the average *β* value in controls at each probe site. We considered a probe as differentially methylated when the absolute value of the difference in *β* value (|Δ*β*|) between two groups was above 0.2 and the false discovery rate (FDR) was below 1%. Detailed methods are available in Additional file 1: Supplementary document.

## Additional files

Additional file 1: Supplementary document. (DOCX 23 kb)

Additional file 2: **Figure S1.** Combination of the results of aCGH and schematic diagram of PWS/AS region. (PPTX 112 kb)

Additional file 3: **Table S1.** Probes on a custom-built oligo-microarray. (XLSX 1717 kb)

Additional file 4: **Table S2.** Probe list of group 1. (XLSX 102 kb)

Additional file 5: **Table S3.** Probe list of group 2. (XLSX 83 kb)

Additional file 6: **Table S4.** Methylation values of group 1 probes. (XLSX 674 kb)

Additional file 7: **Table S5.** Methylation values of group 2 probes. (XLSX 459 kb)

Additional file 8: **Figure S2.** Family trees of cases with microdeletions enrolled in this study. (PPTX 40 kb)

Additional file 9: **Figure S3.** Algorithm for HM450k data processing. (PPTX 46 kb)

## Abbreviations

116HG: 116 host genes
aCGH: Array-based comparative genomic hybridization
AS: Angelman syndrome
HM450k: Infinium HumanMethylation450 BeadChip
IC: Imprinting center
ICR: Imprinting control region
iDMRs: Imprinted differentially methylated regions
LD: Large deletions
MS-MLPA: Methylation-specific multiplex ligation-dependent probe amplification
PWS: Prader-Willi syndrome
sno-lncRNAs: Long non-coding RNAs
SRO: Smallest region of overlap
