# Supplementary material for: Exploring the unique function of imprinting control centers in the PWS/AS-responsible region: finding from array-based methylation analysis in cases with variously sized microdeletions
Source: Clin Epigenetics. 2019 Feb 28;11:36. doi: 10.1186/s13148-019-0633-1 (PMC6396496; doi:10.1186/s13148-019-0633-1)
Supplement: Supplementary file 1 — Supplementary document. (DOCX 23 kb) [file 13148_2019_633_MOESM1_ESM.docx]

**Supplementary Document**

**Clinical manifestations of cases with microdeletions**

Case 1 was a boy who was suspected as having Prader-Willi syndrome (PWS) because of severe hypotonia, poor sucking, facial appearance, and cryptorchidism at two months of age. Hypopigmentation was not noted. The molecular diagnosis was PWS due to a deletion involving AS-IC and PWS-IC and hypermethylation at the PWS-IC detected by Methylation specific multiplex ligation-dependent probe amplification (MS-MLPA). MS-MLPA was performed using the samples of his parents and revealed that the father (Case 2) had the same deletion with hypomethylation at the PWS-IC.

Case 3 was a girl who showed mild hypotonia and developmental delay during infancy and hyperphagia followed by morbid obesity from around 3 years of age. PWS was suspected. Her facial appearance was consistent with PWS. However, she did not present with small hands and feet, growth retardation, or hypopigmentation. The molecular diagnosis was PWS due to a deletion involving the *SNORD116s* cluster without aberrant methylation at the PWS-IC. Her parents did not have that deletion.

Cases 4 and 5 were boys who showed developmental delay during infancy. A happy demeanor that included frequent laughing, smiling, and excitability were noted as they grew up. They had a deletion in AS-IC and hypomethylation at the promoter region of *SNRPN*; Angelman syndrome (AS) was the diagnosis. The parents of Case 4 had no deletion at AS-IC. However, the healthy mother (Case 6) of Case 5 had a deletion in AS-IC and hypermethylation at the promoter region of *SNRPN* and was therefore a carrier of the AS-IC deletion.

Cases with PWS or AS due to conventional large deletions (LD) in 15q11–13 underwent molecular analysis using MS-MLPA. They presented with typical PWS or AS phenotypes.

**Procedure of methylation analysis using HM450k methylation array**

A 750 ng gDNA sample extracted from peripheral blood of cases and controls was bisulfite converted using the EZ DNA Methylation kit (Zymo Research, Orange). Then, the bisulfite-converted gDNA was amplified, fragmented, and hybridized using the Infinium HumanMethylation450 BeadChip (HM450k, Illumina, Inc), and scanned by the Illumina^®^ iScan.

For Cases 1–6 with microdeletions, two aliquots from single gDNA samples were independently labeled, bisulfite treated, and analyzed by the HM450K array as technical replicates, followed by subsequent data analysis. Other samples including PWS or AS with conventional large deletions and normal control subjects were analyzed without replication.

**Algorithm of HM450k data pre-processing**

The raw ‘.idat’ files were analyzed using the ‘ChAMP’ R package version 1.10.0 [1]. For filtering out problematic probes, we applied the default condition of the ‘champ.load’ function for loading ‘.idat’ data. Briefly, we removed probes with a detection *p*-value above 0.01, probes with bead count below 3 beads, probes on sex chromosomes, probes containing SNP, cross-reacting probes, and probes with low success rate (missing in >95% of the samples). We applied the Beta MIxture Quantile dilation (BMIQ) methods for normalization [2].

**Detailed methods for detecting CpG sites and regions with aberrant methylation**

To detect probes with significantly different methylation status between two groups, we utilized the ‘champ.MVP’ function in ChAMP. Because the groups to be compared using the ‘champ.MVP’ function must consist of more than one sample, we utilized replicate data obtained from one case with microdeletion for that comparison using ChAMP. The methylation level at each probe was represented by β-values ranging from 0 (completely unmethylated) to 1 (completely methylated). The differences in DNA methylation (Δβ) were calculated by subtraction of the β-value of each case from the average of β-values of controls at each probe site. We considered a probe as differentially methylated when the absolute value of the difference in beta values (|Δβ|) between two groups was above 0.2 and the false discovery rate (FDR) using Benjamini-Hochberg [3] was below 1%.

We also defined ‘differentially methylated regions (DMR) with aberrant methylation pattern if the DMR contained more than 60% of the total number of analyzed probes showing ‘differentially methylated’ between two groups. All statistical tests were conducted using R version 3.2.0 (www.r-project.org).

**Abbreviation list for this supplementary document**

PWS, Prader-Willi syndrome; AS, Angelman syndrome; DMR, differentially methylated region; LD, large deletion; BP, breakpoint; MS-MLPA, Methylation specific

multiplex ligation-dependent probe amplification; HM450k, Infinium HumanMethylation450 BeadChip; BMIQ, Beta MIxture Quantile dilation; FDR, false discovery rate; IC, imprinting center

**Reference**s

1. Morris TJ, Butcher LM, Feber A, Teschendorff AE, Chakravarthy AR., Wojdacz TK, et al. (2014). ChAMP: 450k Chip Analysis Methylation Pipeline. Bioinformatics. 2014;30;428–430. http://doi: 10.1093/bioinformatics/btt684.
2. Teschendorff AE, Marabita F, Lechner M, Bartlett T, Tegner J, Gomez-Cabrero D, et al. A beta-mixture quantile normalization method for correcting probe design bias in Illumina Infinium 450 k DNA methylation data. Bioinformatics. 2013;29,189–196. doi: 10.1093/bioinformatics/bts680.
3. Benjamini Y, Hochberg Y. Controlling the False Discovery Rate: a Practical and Powerful Approach to Multiple Testing. J R Statist Soc ser B. 1995;57;289–300.
