# Supplementary material for: Exploring the unique function of imprinting control centers in the PWS/AS-responsible region: finding from array-based methylation analysis in cases with variously sized microdeletions
Source: Clin Epigenetics. 2019 Feb 28;11:36. doi: 10.1186/s13148-019-0633-1 (PMC6396496; doi:10.1186/s13148-019-0633-1)
Supplement: Supplementary file 2 — Figure S1. Combination of the results of aCGH and schematic diagram of PWS/AS region. (PPTX 112 kb) [file 13148_2019_633_MOESM2_ESM.pptx]

## Slide 1
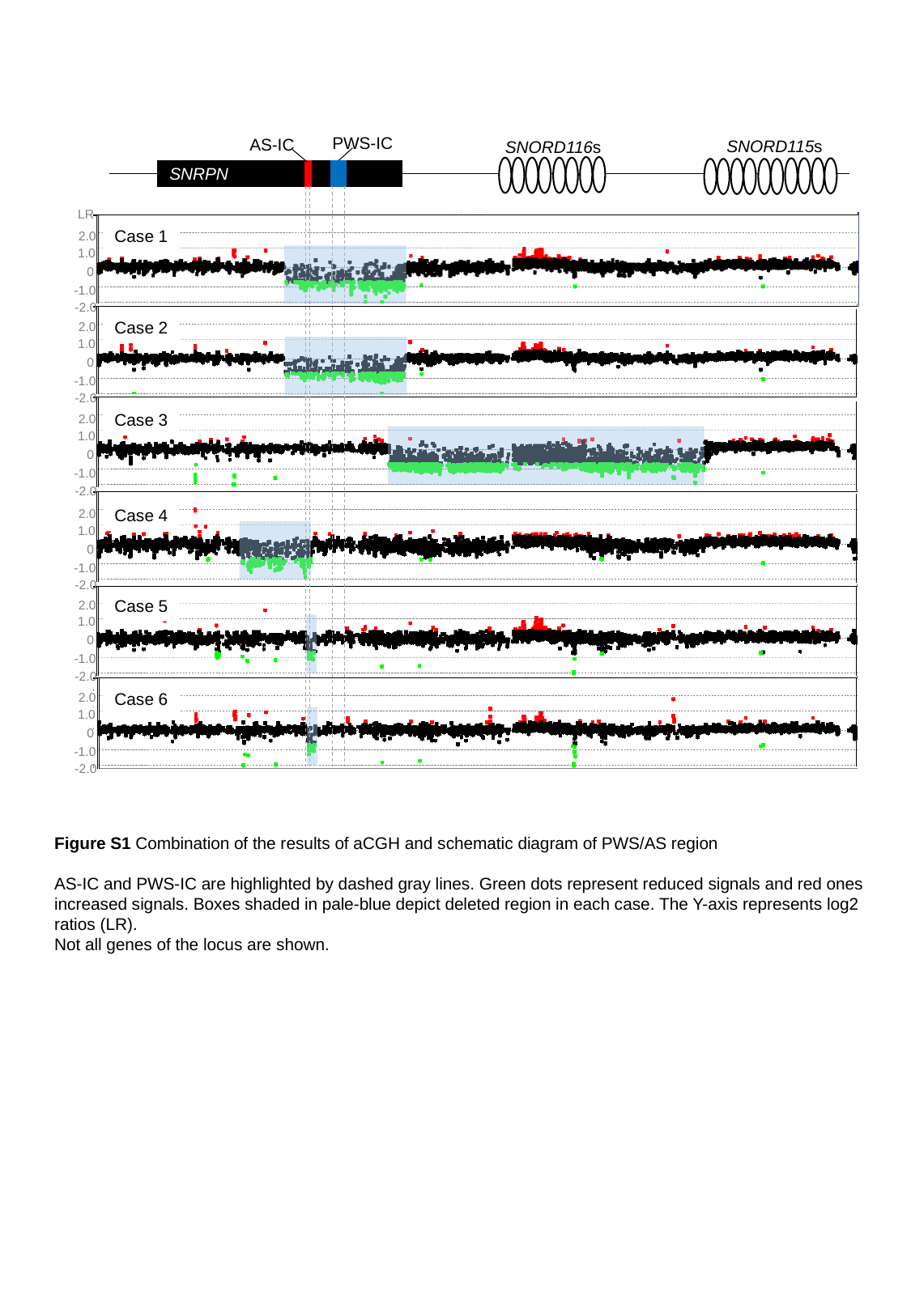

PWS-IC
AS-IC
SNRPN
SNORD115s
SNORD116s
LR
Case 1
2.0
1.0
0
-1.0
-2.0
Case 2
2.0
1.0
0
-1.0
-2.0
Case 3
2.0
1.0
0
-1.0
-2.0
Case 4
2.0
1.0
0
-1.0
-2.0
Case 5
2.0
1.0
0
-1.0
-2.0
Case 6
2.0
1.0
0
-1.0
-2.0
Figure S1 Combination of the results of aCGH and schematic diagram of PWS/AS region
AS-IC and PWS-IC are highlighted by dashed gray lines. Green dots represent reduced signals and red ones increased signals. Boxes shaded in pale-blue depict deleted region in each case. The Y-axis represents log2 ratios (LR).
Not all genes of the locus are shown.
