# Supplementary material for: Exploring the unique function of imprinting control centers in the PWS/AS-responsible region: finding from array-based methylation analysis in cases with variously sized microdeletions
Source: Clin Epigenetics. 2019 Feb 28;11:36. doi: 10.1186/s13148-019-0633-1 (PMC6396496; doi:10.1186/s13148-019-0633-1)
Supplement: Supplementary file 8 — Figure S2. Family trees of cases with microdeletions enrolled in this study. (PPTX 40 kb) [file 13148_2019_633_MOESM8_ESM.pptx]

## Slide 1
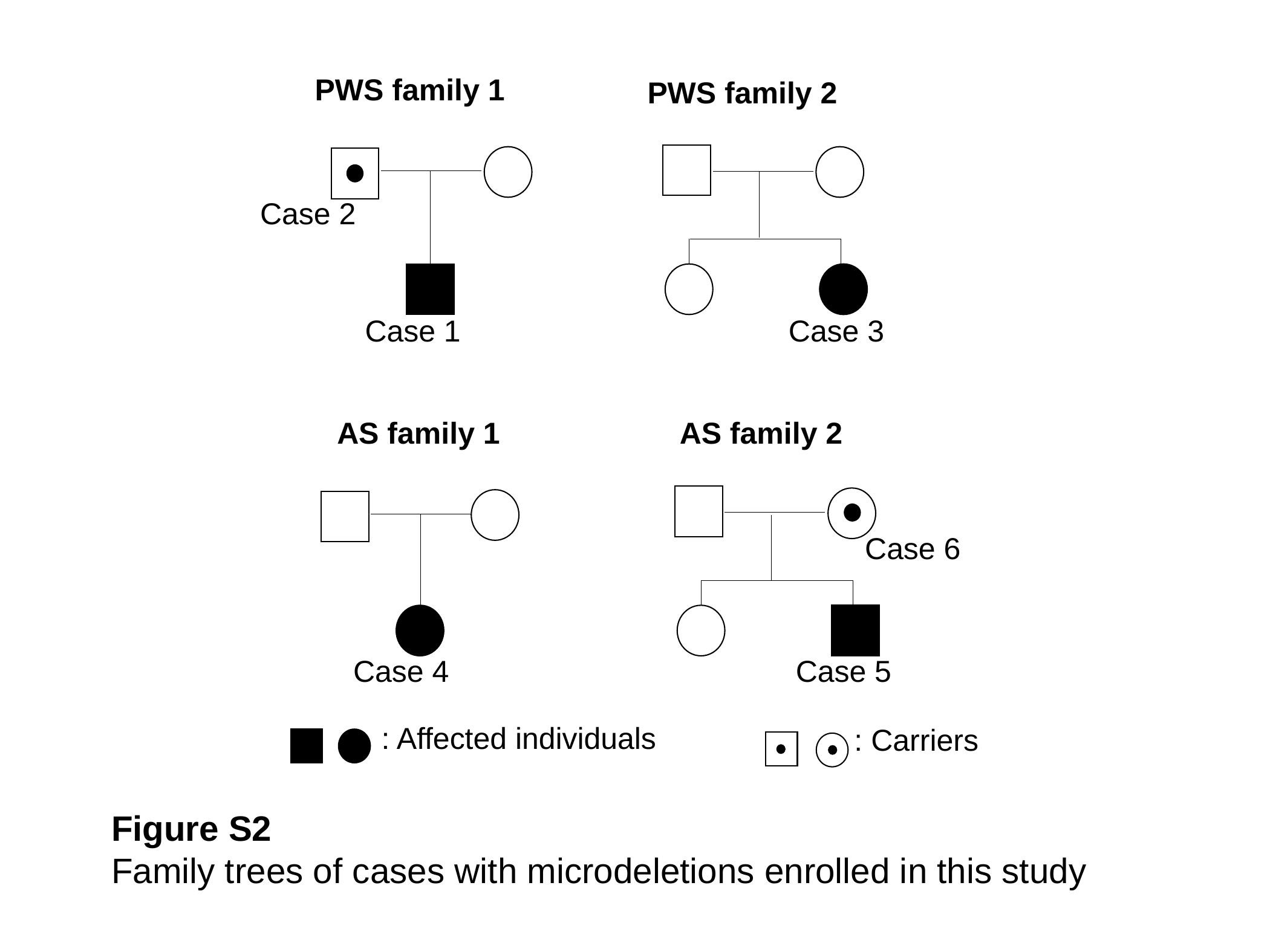

PWS family 1
PWS family 2
Case 2
Case 1
Case 3
AS family 1
AS family 2
Case 6
Case 4
Case 5
: Affected individuals
: Carriers
Figure S2
Family trees of cases with microdeletions enrolled in this study
