# Supplementary material for: Exploring the unique function of imprinting control centers in the PWS/AS-responsible region: finding from array-based methylation analysis in cases with variously sized microdeletions
Source: Clin Epigenetics. 2019 Feb 28;11:36. doi: 10.1186/s13148-019-0633-1 (PMC6396496; doi:10.1186/s13148-019-0633-1)
Supplement: Supplementary file 9 — Figure S3. Algorithm for HM450k data processing. (PPTX 46 kb) [file 13148_2019_633_MOESM9_ESM.pptx]

## Slide 1
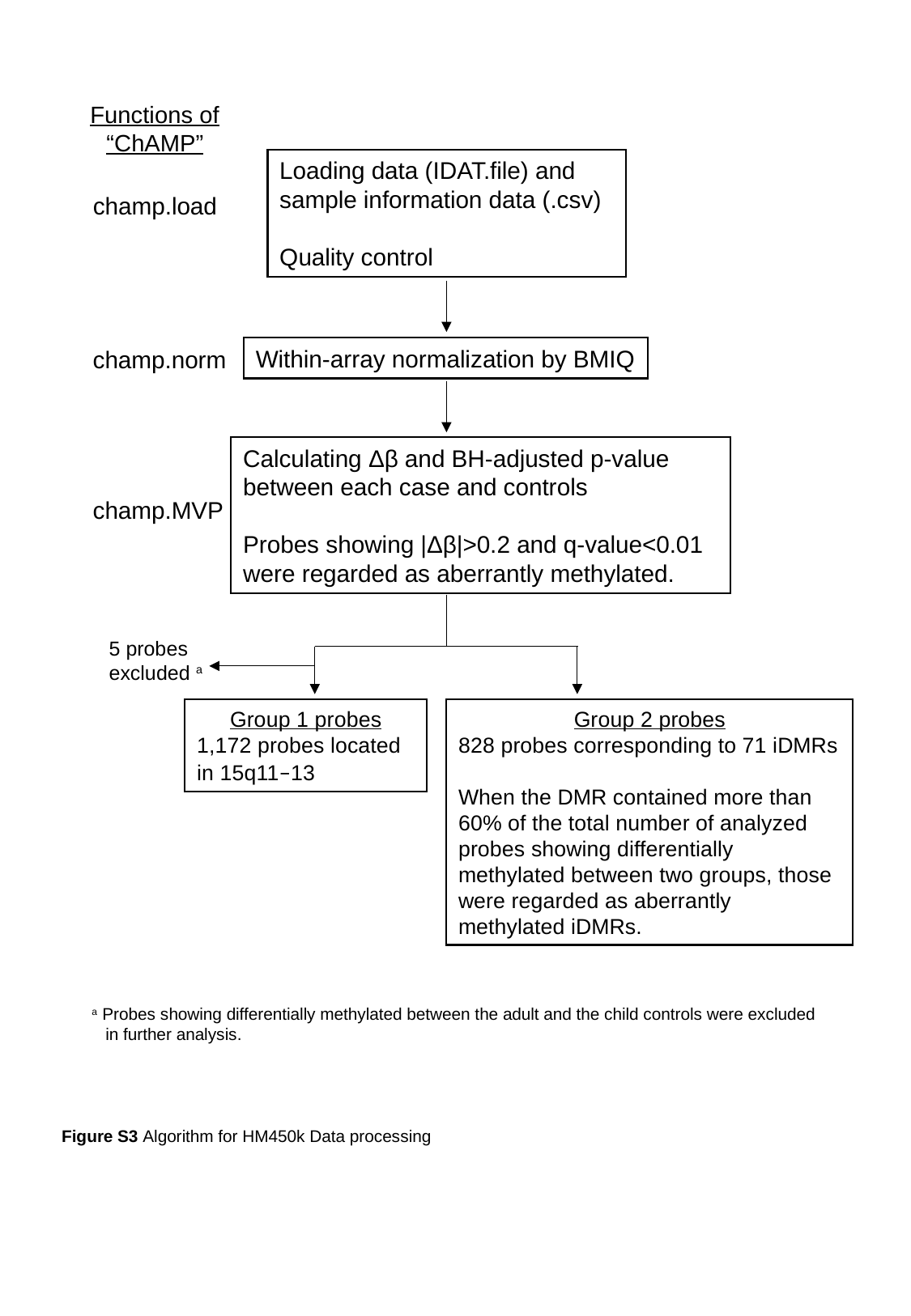

Functions of “ChAMP”
Loading data (IDAT.file) and
sample information data (.csv)
Quality control
champ.load
Within-array normalization by BMIQ
champ.norm
Calculating Δβ and BH-adjusted p-value
between each case and controls
Probes showing |Δβ|>0.2 and q-value<0.01 were regarded as aberrantly methylated.
champ.MVP
5 probes excluded a
Group 1 probes
1,172 probes located in 15q11–13
Group 2 probes
828 probes corresponding to 71 iDMRs
When the DMR contained more than 60% of the total number of analyzed probes showing differentially methylated between two groups, those were regarded as aberrantly methylated iDMRs.
a Probes showing differentially methylated between the adult and the child controls were excluded
 in further analysis.
Figure S3 Algorithm for HM450k Data processing
